# PROtein enriched MEDiterranean diet to combat undernutrition and promote healthy neuroCOGnitive ageing in older adults: The PROMED‐COG consortium project

**DOI:** 10.1111/nbu.12571

**Published:** 2022-07-20

**Authors:** Roisin F. O'Neill, Lorraine Brennan, Federica Prinelli, Giuseppe Sergi, Caterina Trevisan, Lisette C. P. G. M. De Groot, Dorothee Volkert, Stefania Maggi, Marianna Noale, Silvia Conti, Fulvio Adorni, Jayne V. Woodside, Michelle C. McKinley, Bernadette McGuinness, Chris Cardwell, Claire T. McEvoy

**Affiliations:** ^1^ Centre for Public Health, Institute for Global Food Security Queen's University Belfast Belfast Northern Ireland; ^2^ School of Agriculture and Food Science, Institute of Food and Health and Conway Institute University College Dublin Dublin Ireland; ^3^ Institute of Biomedical Technologies, Epidemiology Unit, National Research Council (CNR) Segrate Italy; ^4^ Geriatric Unit, Department of Medicine, University of Padova Padova Italy; ^5^ Division of Human Nutrition Wageningen University Wageningen Netherlands; ^6^ Institute for Biomedicine of Aging Friedrich‐Alexander Universität of Erlangen‐Nümberg Nuremberg Germany; ^7^ Neuroscience Institute, National Research Council (CNR) Padova Italy

**Keywords:** mediterranean diet, neurocognitive ageing, physical activity, protein enrichment, undernutrition

## Abstract

Dementia is a major public health challenge owing to its increasing prevalence and recognised impact on disability among older adults. Observational data indicate that weight loss is associated with increased dementia risk of 30%–40% and precedes a diagnosis of cognitive impairment or dementia by at least one decade. Although relatively little is known about the mechanisms of unintentional weight loss in dementia, this provides a window of opportunity to intervene with strategies to counteract undernutrition and delay, or prevent, the onset of dementia. This article provides an overview of the *PROMED‐COG* project and associated work packages. The project aimes to (1) strengthen the epidemiologic evidence to better understand the potential benefits of combating undernutrition for healthy neurocognitive ageing; (2) increase scientific knowledge on the balance between a protein enriched Mediterranean diet (PROMED) and physical exercise to prevent undernutrition and promote healthy neurocognitive ageing, and generate data on mechanistic pathways; (3) stimulate collaboration and capacity building for nutrition and neurocognitive ageing research in Europe; and (4) develop public and practice recommendations to combat undernutrition and promote healthy neurocognitive ageing in older adults. Findings will provide new and critical insights into the role of undernutrition in neurocognitive ageing, how this role can differ by sex, genetic risk and timing of undernutrition exposure, and how modifications of dietary and physical activity behaviour can reduce the burden of undernutrition and neurodegeneration. The research outcomes will be useful to inform policy and practice about the dietary guidelines of older people and provide insight to industry for the development of food‐based solutions to prevent undernutrition.

## INTRODUCTION

The European population is ageing rapidly with 90.5 million older adults (aged 65 years or older) living within the European Union in 2019 and expected to increase to 129.8 million by 2050 (Eurostat, 2020). A key public health challenge of this ageing population is to ensure citizens remain healthy and disability‐free for as long as possible (World Health Organization, 2019). Dementia is a major public health challenge of ageing particularly given its recognised impact on disability among older adults (World Health Organization, 2017). Approximately 9.1 million people are living with dementia in Europe, and this figure is expected to rise to 14.2 million by 2040 (OECD/European Union, 2018).

Undernutrition—defined as a state resulting from inadequate food intake and/or nutrient deficiencies that lead to an altered body composition and weight loss (Cederholm et al., 2015)—is common among older adults with cognitive impairment (Volkert et al., 2015). Alzheimer's Disease International suggests that 20%–45% of community‐dwelling people with dementia experience clinically significant weight loss (Prince et al., 2014). Undernourished dementia patients experience faster functional and cognitive decline and greater risk of hospitalisation and death, compared with those who are adequately nourished (Spaccavento et al., 2009; Soto et al., 2012; Sanders et al., 2018). Systematic review evidence suggests that annual weight loss greater than or equal to 0.5% is associated with increased dementia risk of 30%–40% (Lee et al., 2020). Although it is possible the association may have arisen through reverse causality, the weight loss precedes a diagnosis of cognitive impairment or dementia by at least one decade (Knopman, 2007; LeBlanc et al., 2016), thus potentially providing a window of opportunity to intervene with strategies to counteract undernutrition, and a potential to delay, or prevent, the onset of dementia in later life.

Relatively, little is known about the mechanisms underpinning undernutrition and dementia (Sergi et al., 2013). Undernutrition has been implicated in the pathway from inflammaging to many chronic diseases, including neurodegenerative diseases (Norman et al., 2021). Furthermore, neurodegenerative processes leading to dementia begin years before clinical features become apparent (Blazer et al., 2015) and induce physiological and behavioural changes, beyond normal ageing, that can alter nutritional status. Changes may include but are not limited to altered inflammatory response (McGrattan et al., 2019), dysregulation of satiety and gut hormones (Cai et al., 2012; Ronveaux et al., 2015), loss of appetite (Kimura et al., 2018), decreased nutrient absorption and altered protein and lipid synthesis (Doorduijn et al., 2019), altered physical activity and food preferences, disrupted sleep patterns (Lucey, 2020), loss of olfactory function and taste (Attems et al., 2015; Olofsson et al., 2021) and social isolation (Poey et al., 2017). It is not clear how these factors might interact to cause unintentional weight loss, whether they are influenced by sex or genetic differences and whether preventing unintentional weight loss can prevent cognitive decline. For reasons not yet clear, apolipoprotein E‐e4 allele (ApoE ε4), the main genetic risk factor for Alzheimer's disease (AD), may contribute to weight loss, especially in women (Vanhanen et al., 2001; Ando et al., 2022). It is possible that ApoE ε4 contributes to accelerated hypothalamic degeneration which can have an adverse impact on leptin signalling, appetite, energy metabolism and food intake (Ando et al., 2022). The reason for sex differences in observed relations between ApoE ε4 is not known, but an interaction effect between sex hormone oestrogen and APOE‐ε4 on cognitive decline has been reported (Ando et al., 2022).

Undernutrition has been linked to accelerated brain atrophy in regions vulnerable to AD (Jimenez et al., 2017) suggesting a pathophysiological relationship between weight loss and cognitive decline (Lee et al., 2020), but only a few neuroimaging studies are available and findings are limited by variation in adjustment for confounders, such as diet, genetic risk, physical activity and medication use. As cognitive impairment progresses, deterioration in cognitive abilities can also affect the ability to shop, cook, prepare and eat meals and engage in physical activity, which can have a deleterious effect on nutritional status (Prince et al., 2014). There is a need to understand the links between undernutrition and cognitive decline, from the earliest to the advanced stages of cognitive impairment to uncover potential mechanisms and inform preventive strategies.

Treatment of undernutrition by protein and energy supplementation has shown some benefit on bodyweight but no convincing effect on cognitive decline, which could be explained by the small number of studies conducted and the weak methodological quality of available data (Allen et al., 2013; Volkert et al., 2015; Correa‐Pérez et al., 2019). Since prior nutrition studies have focused on undernourished dementia patients (Lauque et al., 2004; Pivi et al., 2011; Vicente De Sousa et al., 2017), it is uncertain whether addressing undernutrition can protect against the onset of cognitive impairment. Preliminary data among undernourished, cognitively healthy adults, showed improved cognitive function in response to a 6 months personalised dietetic intervention (Endevelt et al., 2011) and following 6 months consumption of a nutrient‐rich drink (Wouters‐Wesseling et al., 2005). While data lend some support to the hypothesis that improved diet and nutritional status counteracts cognitive decline in older, undernourished adults, more nutrition trials are required to confirm these findings.

## THE PROMED‐COG CONSORTIUM

**FIGURE 1:**
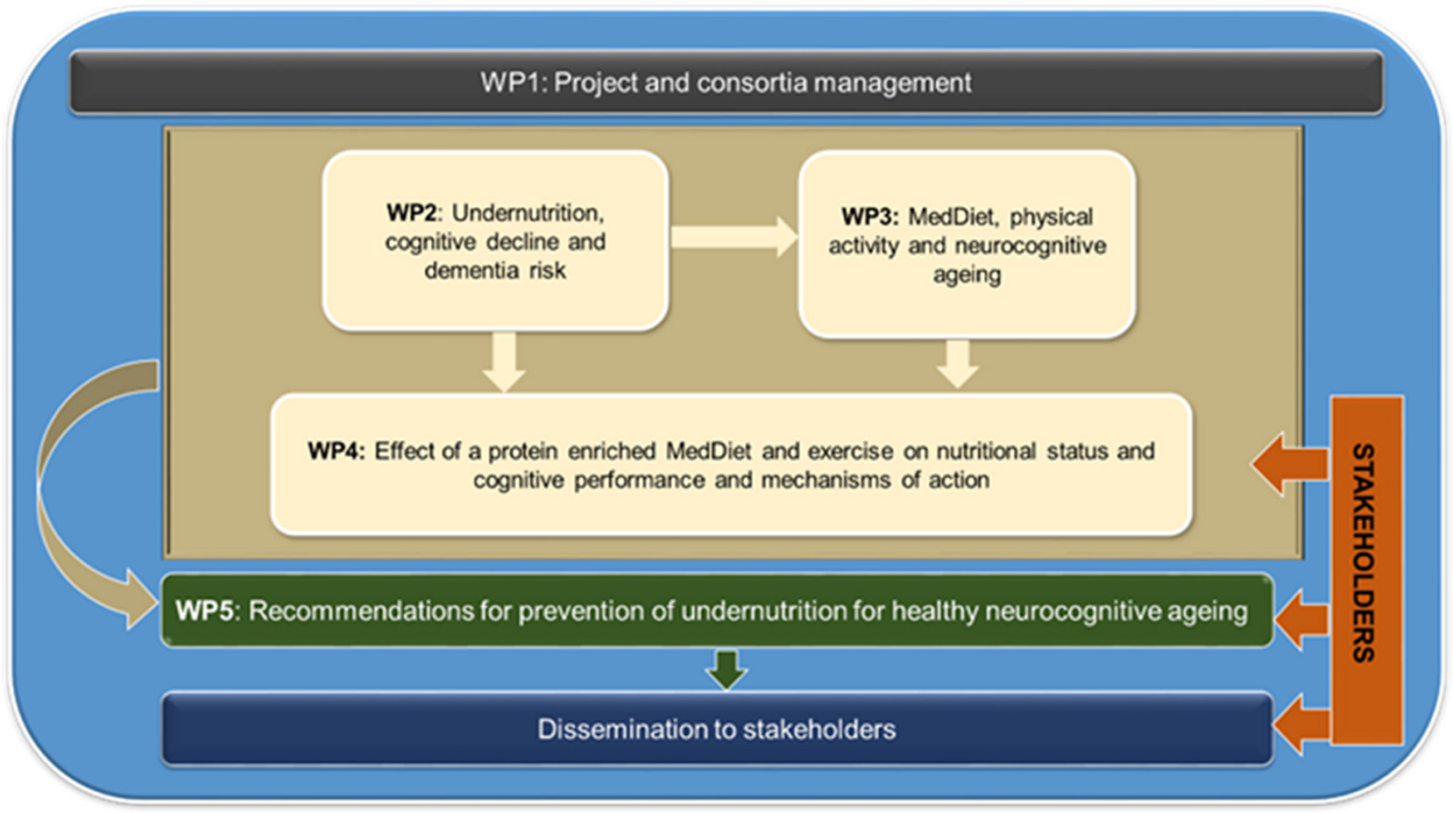
Overview of the PROMED‐COG work packages. The PROMED‐COG consortium comprises four partners and two collaborators across leading European Institutions (see Table 1). Other external stakeholder partners in the project include dietitians and geriatricians (UK), public and patient involvement (PPI) representatives (UK), the European Federation of the Association of Dietitians (EFAD), the British Dietetic Association Northern Ireland and the European Nutrition for Health Alliance (ENHA), to represent broader professional societies and patients groups involved in prevention of undernutrition in older age

The *PROtein enriched MEDiterranean diet to combat undernutrition and promote healthy neuroCOGnitive ageing* (*PROMED‐COG*) transnational consortium project is funded under the European Horizon 2020 Joint Programming Initiative ‘a Healthy Diet for a Healthy Life’ (JPI HDHL) and the ERA‐NET Cofund ERA‐HDHL, specifically via the PREVNUT call for development of targeted nutrition for prevention of undernutrition for older adults. *PROMED‐COG* brings together a multidisciplinary scientific team from the UK, Ireland, Italy, The Netherlands and Germany as well as external stakeholders to represent the broader professional societies and patient groups involved in nutrition for public health outlined in Figure 1 and Table 1.

**TABLE 1:** Overview of PROMED‐COG principal research partners

Research partner	Institute, country	Primary role in PROMED‐COG	Area of expertise	Funder
Dr Claire McEvoy	Queen's University Belfast, UK	Project Co‐Ordinator Leads the PROMED‐EX randomised controlled trial (WP4)	Nutrition and cognitive ageing	UK Research & Innovation: Biotechnology and Biological Sciences Research Council and Medical Research Council
Professor Lorraine Brennan	University College Dublin, Ireland	Leads the metabolomic analyses (WPs 3–4)	Dietary biomarkers and metabolomics	Health Research Board
Dr Federica Prinelli	National Research Council (CNR), Italy	Leads the epidemiologic analyses (WP 3)	Nutritional epidemiology of ageing	Italian Ministry of Universities and Research
Professor Guiseppi Sergi	University of Padova, Italy	Leads the epidemiologic analyses (WP 2)	Nutrition and Geriatric medicine	Italian Ministry of Universities and Research
Professor Lisette de Groot	Wageningen University, The Netherlands	Scientific advisor for the overall project	Nutrition and ageing	‐‐
Professor Dorothee Volkert	Friedrich‐Alexander Universität of Erlangen‐Nümberg, Germany	Scientific advisor for the overall project	Clinical nutrition in older persons	‐‐

The primary objectives of *PROMED‐COG* are to:

1. Strengthen epidemiologic evidence to better understand the potential benefits of combating undernutrition for healthy neurocognitive ageing.
2. Increase scientific knowledge on the balance between a protein enriched Mediterranean diet (PROMED) and physical exercise to prevent undernutrition and promote healthy neurocognitive ageing and generate data on mechanistic pathways.
3. Stimulate collaboration and capacity building for nutrition and neurocognitive ageing research in Europe.
4. Develop public and practice recommendations to combat undernutrition and promote healthy neurocognitive ageing in older adults.

Project objectives will be delivered by five cross‐cutting work packages (WPs) that combine both epidemiological and intervention studies to address gaps in the scientific knowledge and engage stakeholders to translate findings into population dietary and lifestyle recommendations for healthy neurocognitive ageing. *PROMED‐COG* project WPs are summarised in Figure 1 and discussed in more detail below.

## WP1: PROJECT AND CONSORTIA MANAGEMENT

The project is coordinated by Queen's University Belfast, working in close collaboration with the other scientific partners to ensure the project runs smoothly. The collaboration agreement and data management plan have been implemented so that scientific excellence and knowledge sharing are maintained throughout the project and all outputs and deliverables are met. This WP will also develop project communications (e.g. media release; project website) and will expand the current scientific, stakeholder and public and patient involvement (PPI) representatives' network. Guided by best practice INVOLVE guidelines (www.invo.org.uk), we will recruit a PPI group comprised of older people with lived experience of subjective cognitive decline and/or undernutrition to co‐produce the PROMED‐EX Trial outlined in WP4 and at least one PPI representative will be invited onto the Trial Steering Committee. A further important part of WP1 is to build research capacity for nutrition and cognitive ageing research by training and mentoring early‐stage researchers (aiming for at least two PhD students and two postdoctoral researchers) in the field of nutrition and cognitive health.

## WP2: UNDERNUTRITION, COGNITIVE DECLINE AND DEMENTIA RISK

There is limited evidence as to whether undernutrition affects the rate of cognitive decline and the onset of dementia, or whether differences exist between men and women. *PROMED‐COG* will exploit data from existing cohort studies to provide improved estimates of exposure to undernutrition on cognitive decline and dementia and identify determinants of weight loss and undernutrition in the population. Moreover, possible modifying factors on the association between undernutrition and cognitive deterioration will also be evaluated.

WP2 is led by partners from the University of Padova, Italy, and will pool data from three epidemiological Italian cohorts that are considered compatible on the criterion of adequate quality exposure and outcomes (Lauque et al., 2004; Pivi et al., 2011; Salva et al., 2011). The *Italian Longitudinal Study of Ageing (ILSA)* is a large‐scale, multi‐centre, longitudinal study on the development of age‐related disease in *n* = 5628 randomly selected adults aged 65–84 years at baseline and followed for 8 years (Maggi et al., 1994). The *Progetto Veneto Anziani (Pro.V.A.)* is a longitudinal study of determinants of disability in an age‐ and sex‐stratified random sample of *n* = 3099 mainly community‐dwelling (96%) adults aged ≥65 years followed for 7 years (Corti MC et al., 2002).The Italian *Bollate Eye Study (BEST)* is a longitudinal study in *n* = 1604 dementia free community‐dwelling individuals from the Lombardy Region (Northern Italy) (aged 40–74 years at enrolment in 1992–1993) and followed over 20 years (Prinelli et al., 2018). Combining *ILSA*, *Pro.V.A* and *BEST* will create a large cohort ranging from mid‐to‐later life with a sample size up to *n* = 10 331 (age range 42–103 years; 52% female).

Retrospective data harmonisation will be performed in accordance with guidelines (Fortier et al., 2017) and used to derive target exposure (undernutrition) and outcome (cognition) variables for the pooled analysis. Undernutrition will be operationalised in two ways: (i) Global Leadership Initiative on Malnutrition (GLIM) criteria for the presence of one or more phenotypic criteria (including weight loss, low body mass index or reduced muscle mass), and at least one etiological criteria (including reduced food intake, inflammation or disease burden) (Cederholm et al., 2015) and (ii) percentage weight loss calculated as weight change from baseline. The outcomes of interest are as follows: (i) Cognitive decline using Mini‐Mental State Examination score (MMSE); (ii) cognitive impairment (defined as one SD less than the population mean MMSE); and iii) incident dementia based on expert consensus to define dementia cases from cohort data including both clinical diagnosis of dementia using, for example DSM III‐R criteria, and health record linkage data by applying a specific algorithm (Prinelli et al., 2018 *)*.

## WP3: MEDITERRANEAN DIET, PHYSICAL ACTIVITY AND NEUROCOGNITIVE AGEING

The Mediterranean Diet (MedDiet) and physical activity (PA) have been independently associated with a decreased risk of Alzheimer's disease (Livingston et al., 2017; Scarmeas et al., 2006; Anastasiou et al., 2018; Limongi et al., 2020), and their combination may have the strongest effect on reducing disease risk (Richard et al., 2013). However, data are limited by the few studies available and further research is needed to assess the potentially additive effects for diet and PA on neurodegeneration and end‐stage dementia. Neuroimaging biomarkers, such as structural MRI, provide sensitive measures of brain health and may help to elucidate potential mechanisms of modifiable behaviours on cognitive decline. To date, the combined effect of MedDiet and PA on brain structure is unknown. In addition, the biological mechanisms underlying associations between diet and neurocognition have been largely unexplored.

WP3 is led by National Research Council (CNR), Italy, and will address these scientific gaps. WP3 will firstly estimate the longitudinal associations between combined MedDiet and PA (MedEx), cognitive decline and risk of dementia using the pooled dataset from *ILSA*, *PRO.V.A* and *BEST* described in WP2 above, that also include self‐reported dietary intake and PA (minutes/week) across the three cohorts. Secondly, WP3 will estimate independent cross‐sectional associations between MedEx and neuroimaging measures (including measures of total, grey matter, white matter volume and white matter lesions) and determine what factors may influence associations, for example socio‐demographic, body composition, inflammatory markers, cardiometabolic risk factors and depression. Data will be drawn from subsamples of two Italian cohorts, *PRO.VA* (*n* = 808) (Corti et al., 2002) and the cross‐sectional *NutBrain study* (www.nutbrain.it/study‐project) (*n* = 150) (Prinelli et al., 2020), that have complete data on ApoE ε4 genotyping and structural brain magnetic resonance imaging (MRI) (pooled cohort *n* = 958; age range 65–95 years; 58% female). WP3 will also explore the mechanistic pathways involved in diet‐associated neurocognition, hypothesising that a MedDiet enhances cognitive function and protects against dementia via favourable effects on brain structures, inflammatory markers and body composition, mediated by favourable metabolomic profiles (see WP4), and better nutrient intake. Inflammatory markers (C‐reactive protein and interleukin 6 [IL‐6]) and metabolomic analysis will be performed on blood samples from the *NutBrain* cohort to determine how the MedDiet might influence circulating metabolites and how the metabolites could mediate the associations between diet and neurocognitive measures.

## WP4: THE PROMED‐EX TRIAL—EFFECT OF A PROTEIN ENRICHED MEDITERRANEAN DIET AND EXERCISE INTERVENTION ON NUTRITIONAL STATUS AND COGNITIVE PERFORMANCE

It is critical to understand the role of lifestyle interventions for preventing undernutrition and promoting healthy neurocognitive ageing, particularly when implemented at an early stage of disease and in ‘high risk’ populations. The MedDiet is highly palatable and nutrient dense and, in this regard, may be both neuroprotective and helpful in preventing undernutrition. The MedDiet has been associated with reduced risk of nutritional deficiencies (Castro‐Quezada et al., 2014), while the moderate fat content of the diet enhances the absorption of micronutrients, especially the fat‐soluble vitamins A, D, E and K (Tosti et al., 2018) that are important for cognitive health during ageing (Prinelli et al., 2019). Adequate protein intake is also a key nutritional factor for prevention of undernutrition. Older adults have an increased requirement for dietary protein (1.2–1.5 g/kg bodyweight/day) to prevent and ameliorate sarcopenia, defined as loss of muscle mass and function (Bauer et al., 2013). Furthermore, exercise can stimulate protein synthesis (Trommelen et al., 2019) and enhance muscle mass, strength and functional performance in older adults (McGlory et al., 2019) making it an attractive target for prevention of undernutrition. An optimised protein MedDiet may induce synergistic effects on nutritional status and cognition when combined with exercise, but this has not yet been evaluated.

WP4, led by Queen's University Belfast, UK, will test the effect of a protein optimised MedDiet (PROMED), or PROMED + exercise (PROMED‐EX) intervention, on nutritional and cognitive status in older people at risk of undernutrition and cognitive decline, and explore potential mechanisms underlying intervention responsiveness. A single‐blind, parallel group randomised controlled trial (RCT) will be conducted (ref: Clinical Trials.gov NCT 05166564) where *n* = 105 eligible participants will be block randomised into one of three groups: *Group 1* (PROMED [*n* = 35]) will receive personalised dietary advice, written education resources (including recipes and meal plans), a home‐delivered supply of key PROMED foods, self‐monitoring tools to facilitate adherence to the intervention and weekly telephone support; *Group 2* (PROMED‐EX [*n* = 35]) will receive the PROMED intervention described above—plus an individually tailored, home‐based exercise programme; and *Group 3* (Control [*n* = 35]) will receive a standard care diet sheet. The active intervention will be delivered over 3 months with extended follow‐up to 6 months (i.e. 3 months after the intervention has ceased).

The PROMED‐EX trial will recruit eligible community‐dwelling individuals, aged ≥60 years old who are at high nutritional risk (based on the Mini Nutritional Assessment [MNA]) and have self‐reported decline in cognition. Those who are malnourished, receiving artificial nutritional support, and have major dietary restriction or diagnosis of cognitive impairment/dementia will be excluded. The primary outcome is between‐group difference in MNA score at 3 and 6 months from baseline. Secondary outcomes will include between‐group difference at 3 and 6 months in cognitive function, diet quality, nutritional biomarkers, PA, body composition, physical performance and psychosocial health. WP4 will also explore the acceptability and tolerance of the interventions as well as ability to change diet and exercise behaviours.

## EXPLORING MECHANISTIC PATHWAYS

It is paramount to establish mechanistic pathways by which diet and exercise may act to inform evidence‐based recommendations and pave the way for innovative food solutions to prevent undernutrition. Across studies, we will use metabolomic and biomarker assessments to uncover biological mechanisms underlying how diet can influence nutrition status and neurocognitive ageing, and how PA interacts with nutrition to influence these processes. In WP4, change in metabolomic profile, lipid profile, inflammatory and metabolic biomarkers and metabolic signals will be investigated using a blood sample collected at each study time point.

University College Dublin, Ireland, will lead the metabolomic analyses for the project. Metabolomics will be performed using a combination of NMR and LC–MS‐based approaches (Macias et al., 2019). A detailed metabolite profile from 7 compound classes including amino acids, biogenic amines, hexoses, acylcarnitines, lysophosphatidylcholines, glycerophospholipids and sphingolipids will be measured. We will explore metabolites in relation to metabolic and inflammatory pathways, for example several of the lipids measured are linked to inflammatory pathways. Combining both targeted and untargeted approaches from different platforms for metabolomics will enhance the coverage of the metabolome in the project (Brennan et al., 2021).

## WP5: DEVELOP PUBLIC AND PRACTICE RECOMMENDATIONS TO COMBAT UNDER NUTRITION AND PROMOTE HEALTHY NEUROCOGNITIVE AGEING IN OLDER ADULTS

The overarching goal of the *PROMED‐COG* project is to utilise the data generated to develop key public and practice recommendations to prevent undernutrition and promote healthy neurocognitive ageing in European adults. A scientific report will be produced describing the key findings and learning from the project and the impact of these findings on the health of older European adults. This objective will focus on raising awareness, sharing knowledge and improving dialogue between stakeholders involved in the areas of healthy ageing, preventable malnutrition and cognitive decline including academics, healthcare providers, public health personnel and the target population of older at‐risk adults.

At least one knowledge exchange workshop will be held (with partners and stakeholders) to develop: (1) the key public health messages arising from this work; (2) practice recommendations for professionals; and (3) areas for future research and a dissemination strategy to report findings. We anticipate that findings will be published in academic journals, policy briefs and reports for practitioners and external shareholders.

## DATA MANAGEMENT

All research is compliant with ethical principles and applicable international, EU and national law. Data from the project will be handled, computerised and stored in accordance with the European General Data Protection Regulation (EU) 2016/679 (GDPR) (https://gdpr‐info.eu/) or the UK GDPR (https://ico.org.uk/for‐organisations/guide‐to‐data‐protection/guide‐to‐the‐general‐data‐protection‐regulation‐gdpr/) where applicable. *PROMED‐COG* partners have agreed a data management plan in compliance with FAIR [Findable, Accessible, Interoperable and Re‐usable] data management principles, to which all researchers involved in the project will adhere.

In brief, project *findability* has been optimised by creating a project website (https://www.promed‐cog.com) to communicate research activities across a broad range of audiences. The PROMED‐EX trial has been registered on the ClinicalTrials.gov database (NCT05166564). Furthermore, the study description and variable metadata catalogues will be published during the project and will be discoverable in open access repositories, for example Zenodo (https://zenodo.org/), partner institutions repositories, for example QUB Pure Research portal, and published in the JPIHDHL Meta Database (http://www.jpihdhl.eu) knowledge transfer platform to ensure visibility to the wider research community. *Accessibility* of research will be maximised via open access publication. Only de‐identified participant data will be published, and participant consent for this will be in place across studies to allow us to share research findings*. Intraoperability* of generated data will be promoted by ensuring metadata files are available via interchangeable statistical file format. Data will be archived and held securely according to local policies, with guaranteed preservation for 10 years to maximise data sharing and *reusability* opportunities. Data sharing and biological sample transfer for the purposes of the proposed analyses will also follow institutional guidelines and Material Transfer Agreements where applicable. Existing and new data collected within the project will be anonymised and archived in password‐protected study databases.

## INNOVATION AND NOVELTY OF PROMED‐COG

The *PROMED‐COG* project is innovative and novel in scope for several reasons:

1. It offers a novel strategy to understand the cumulative risks of undernutrition for cognitive decline and dementia particularly as processes leading to cognitive decline and dementia may begin, and be affected by, undernutrition earlier in life, yet studies to date have focused on the effects of weight loss at end‐stage disease. The data from the pooling of these epidemiological cohorts will enable us to examine the cumulative risk of undernutrition from the earliest to the latest stages of disease and examine whether the associations differ by sex and genetic risk.
2. The unique focus on diet and physical activity patterns, rather than individual foods or behaviours, allows a ‘real‐world’ evaluation of the potential additive effects of healthy lifestyle behaviours on a range of neurocognitive ageing measures, including neuroimaging biomarkers and cognitive performance across several domains. This approach will facilitate effective translation of the scientific knowledge gained from our project into lifestyle recommendations for public health benefit.
3. For the first time, we will test a complex lifestyle intervention to determine whether adopting a protein‐enriched MedDiet can improve both nutritional status and cognition—an important health outcome for older adults—and whether there are further benefits offered by exercise. This methodological approach will complement the planned epidemiological analyses to generate robust evidence to inform public health recommendations and dietary guidelines.
4. There is integration of state‐of‐the‐art metabolomics, metabolic and nutritional biomarkers to elucidate biological mechanisms underlying how an adapted MedDiet can influence nutrition status and neurocognitive ageing, and how PA can interact with nutrition to modify these processes.
5. The linkage of nutrition and ageing researchers across Europe allows sharing of expertise and knowledge in multi‐domain lifestyle interventions biomarker development and assessment, epidemiology, ageing and cognitive function assessment.

## CONCLUSIONS

The *PROMED‐COG* project (2021–2023) aims to combine epidemiological and intervention research to provide new and critical insight into the role of undernutrition in neurocognitive ageing, how these effects can differ by sex, genetic risk and timing of undernutrition exposure, and how modifications of dietary and PA behaviour may act to reduce the burden of undernutrition and neurodegeneration. This is of public health importance given the increase in the ageing population across Europe and the link between undernutrition and dementia risk. The resulting scientific knowledge will be translated into public and practice recommendations to prevent undernutrition for healthy neurocognitive ageing in older European citizens.

## CONFLICT OF INTEREST

There are no conflicts of interest to declare.

## Data Availability

Data sharing not applicable ‐ no new data generated

## References

[nbu12571-bib-0001] Allen, V. , Methven, L. & Gosney, M. (2013) The influence of nutritional supplement drinks on providing adequate calorie and protein intake in older adults with dementia. Journal of Nutrition, Health and Aging, 17(9), 752–755. 10.1007/s12603-013-0364-5 24154647

[nbu12571-bib-0002] Anastasiou, C.A. , Yannakoulia, M. , Kontogianni, K.M.H. , Mamalaki, E. , Dardiotis, E. , Hadjigeorgiou, G. et al. (2018) Mediterranean lifestyle in relation to cognitive health: results from the HELIAD study. Nutrients, 10(10), 1557. 10.3390/nu10101557 PMC6213445 30347812

[nbu12571-bib-0003] Ando, T. , Uchida, K. , Sugimote, T. , Kimura, A. , Saji, N. , Niida, S. & Sakurai, T. (2022) ApoE4 is associated with lower body mass, particularly fat mass, in older women with cognitive impairment. Nutrients, 14, 539. 10.3390/nu14030539 35276898 PMC8838979

[nbu12571-bib-0004] Attems, J. , Walker, L. & Jellinger, K.A. (2015) Olfaction and aging: A mini‐review. Gerontology, 61(6), 485–490. 10.1159/000381619 25968962

[nbu12571-bib-0005] Bauer, J. , Biolo, G. , Cedarholm, T. , Cesari, M. , Cruz‐Jentoft, A.J. , Morley, J.E. et al. (2013) Evidence‐based recommendations for optimal dietary protein intake in older people: a position paper from the prot‐age study group. Journal of the American Medical Directors Association, 14(8), 542–559. 10.1016/j.jamda.2013.05.021 23867520

[nbu12571-bib-0006] Blazer, D.G. , Yaffe, K. & Karlawish, J. (2015) Cognitive aging: A report from the institute of medicine. Journal of the American Medical Association, 313(21), 2121–2122. 10.1001/jama.2015.4380 25875498

[nbu12571-bib-0007] Brennan, L. , Hu, F.B. & Sun, Q. (2021) Metabolomics meets nutritional epidemiology: Harnessing the potential in metabolomics data. Metabolites, 11(10), 709. 10.3390/metabo11100709 34677424 PMC8537466

[nbu12571-bib-0008] Castro‐Quezada, I. , Román‐Viñas, B. & Serra‐Majem, L. (2014) The mediterranean diet and nutritional adequacy: A review. Nutrients, 6(1), 231–248. 10.3390/nu6010231 24394536 PMC3916858

[nbu12571-bib-0009] Cederholm, T. , Bosaeus, I. , Barazonni, R. , Bauer, J. , Van Gossum, A. , Klek, S. et al. (2015) Diagnostic criteria for malnutrition ‐ an ESPEN consensus statement. Clinical Nutrition, 34(3), 335–340. 10.1016/j.clnu.2015.03.001 25799486

[nbu12571-bib-0010] Correa‐Pérez, A. , Abraha, I. , Cherubini, A. , Collinson, A. , Dardevet, D. , de Groot, L.C.P.G.M. et al. (2019) Efficacy of non‐pharmacological interventions to treat malnutrition in older persons: A systematic review and meta‐analysis. The SENATOR project ONTOP series and MaNuEL knowledge hub project. Ageing Research Reviews, 49, 27–48. 10.1016/j.arr.2018.10.011 30391755

[nbu12571-bib-0011] Cai, H. , Cong, W.N. , Ji, S. , Rothman, S. , Maudsley, S. & Martin, B. (2012) Metabolic dysfunction in Alzheimer's disease and related neurodegenerativedisorders. Current Alzheimer Research, 9(1), 5–17. 10.2174/156720512799015064 22329649 PMC4097094

[nbu12571-bib-0012] Corti, M.C. , Guralnik, J.M. , Sartoni, L. , Baggio, G.M.E. , Manzato, E. & Pezzotti, P. (2002) The effect of cardiovascular and osteoarticular diseases on disability in older Italian men and women: rationale, design, and sample characteristics of the Progetto Veneto Anziani (PRO.V.a.) study. Journal of American Geriatric society, 50(9), 1535–1540. 10.1046/j.1532-5415.2002.50409.x 12383151

[nbu12571-bib-0013] Doorduijn AS , van de Rest O , van de Flier W , Visser, M , Visser M , & de van der Schueren MAE (2019) Energy and protein intake of Alzheimer's disease patients compared to cognitively normal controls: Systematic review. Journal of the American Medical Directors Association, 20(1), 14–21. 10.1016/j.jamda.2018.06.019. 30100233

[nbu12571-bib-0014] Endevelt, R. , Lemberger, J. , Bregman, J. , Kowen, G. , Berger‐Fecht, I. , Lander, H. et al. (2011) Intensive dietary intervention by a dietitian as a case manager among community dwelling older adults: The EDIT study. Journal of Nutrition, Health and Aging, 15(8), 624–630. 10.1007/s12603-011-0074-9 21968856

[nbu12571-bib-0015] Eurostat (2020) Ageing Europe ‐ looking at the lives of older people in the EU, Eurrostat. Luxembourg: Publications Office of the European Union. Available at: https://ec.europa.eu/eurostat/web/products‐statistical‐books/‐/ks‐02‐20‐655 [Accessed 15 Jan 2022]

[nbu12571-bib-0016] Fortier, I. , Raina, P. , Van den Heuvel, E.R. , Griffith, L.E. , Craig, C. , Saliba, M. et al. (2017) Maelstrom research guidelines for rigorous retrospective data harmonization. International Journal of Epidemiology, 46(1), 103–115. 10.1093/ije/dyw075 27272186 PMC5407152

[nbu12571-bib-0017] Jimenez, A. , Pegueroles, J. , Carmona‐Iragui, M. , Vilaplana, E. , Montal, V. , Alcolea, D. et al. (2017) Weight loss in the healthy elderly might be a non‐cognitive sign of preclinical Alzheimer's disease. Oncotarget, 8(62), 104706–104716. 10.18632/oncotarget.22218 29285207 PMC5739594

[nbu12571-bib-0018] Kimura, A. , Sugimoto, T. , Niida, S. , Toba, K. & Sakurai, T. (2018) Association between appetite and sarcopenia in patients with mild cognitive impairment and early‐stage Alzheimer's disease: a case‐control study. Frontiers in Nutrition, 5, 1–9. 10.3389/fnut.2018.00128 30619874 PMC6305366

[nbu12571-bib-0019] Knopman, D.S. , Edland, S.D. , Cha, R.H. , Petersen, R.C. & Rocca, W.C. (2007) Incident dementia in women is preceded by weight loss by at least a decade. Neurology, 69(8), 739–746. 10.1212/01.wnl.0000267661.65586.33 17709705

[nbu12571-bib-0020] Lauque, S. , Arnaud‐Battandier, F. , Gillette, S. , Plaze, J.M. , Andrieu, S. , Cantet, C. et al. (2004) Improvement of weight and fat‐free mass with oral nutritional supplementation in patients with Alzheimer's disease at risk of malnutrition: A prospective randomized study. Journal of the American Geriatrics Society, 52(10), 1702–1707. 10.1111/j.1532-5415.2004.52464.x 15450048

[nbu12571-bib-0021] LeBlanc, E.S. , Rizzo, J.H. & Pedula, K. (2016) Weight trajectory over 20 years and likelihood of mild cognitive impairment or dementia among older women. Journal of the American Geriatrics Society, 65, 511–519. 27991654 10.1111/jgs.14552 PMC5685172

[nbu12571-bib-0022] Lee, C.M.Y. , Woodward, M. , Batty, G.D. , Beiser, A.S. , Bell, S. , Berr, C. et al. (2020) Association of anthropometry and weight change with risk of dementia and its major subtypes: A meta‐analysis consisting 2.8 million adults with 57 294 cases of dementia. Obesity Reviews, 21(4), 1–14. 10.1111/obr.12989 PMC7079047 31898862

[nbu12571-bib-0023] Limongi, F. , Siviero, P. , Bozanic, A. , Noale, M. , Veronese, N. & Maggi, S. (2020) The effect of adherence to the Mediterranean diet on late‐life cognitive disorders: A systematic review. Journal of the American Medical Directors Association, 21(10), 1402–1409. 10.1016/j.jamda.2020.08.020 32981667

[nbu12571-bib-0024] Livingston, G. , Sommerlad, A. , Orgeta, V. , Costafreda, S.G. , Huntley, J. , Ames, D. et al. (2017) The lancet international commission on dementia prevention and care. Lancet, 390(10113), 2673–2734. 10.1016/S0140-6736(17)31363-6 28735855

[nbu12571-bib-0025] Lucey, B.P. (2020) It's complicated: the relationship between sleep and Alzheimer's disease in humans. Neurobiology of Disease, 144, 105031. 10.1016/j.nbd.2020.105031 32738506 PMC7484285

[nbu12571-bib-0026] Macias, S. , Kirma, J. , Yilmaz, A. , Moore, S. , McKinley, M. , McKeown, P. et al. (2019) Application of 1H‐NMR metabolomics for the discovery of blood plasma biomarkers of a Mediterranean diet. Metabolites, 9(10), 201. 10.3390/metabo9100201 PMC6836148 31569638

[nbu12571-bib-0027] Maggi, S. , Zucchetto, M. , Grigoletto, F. , Baldereschi, M. , Candelise, C. , Scarpini, E. et al. (1994) The Italian longitudinal study on aging (ILSA): Design and methods. Aging Clinical and Experimental Research, 6(6), 464–473. 10.1007/BF03324279 7748921

[nbu12571-bib-0028] McGlory, C. , van Vliet, S. , Stokes, T. , Mittendorfer, B. & Phillips, S. (2019) The impact of exercise and nutrition on the regulation of skeletal muscle mass. Journal of Physiology, 597(5), 1251–1258. 10.1113/JP275443 30010196 PMC6395419

[nbu12571-bib-0029] McGrattan, A.M. , McGuinness, B. , McKinley, M. , Kee, F. , Passmore, P. , Woodside, J.V. et al. (2019) Diet and inflammation in cognitive ageing and Alzheimer's disease. Current Nutrition Reports, 8(2), 53–65. 10.1007/s13668-019-0271-4 30949921 PMC6486891

[nbu12571-bib-0030] Norman, K. , Haß, U. & Pirlich, M. (2021) Malnutrition in older adults—recent advances and remaining challenges. Nutrients, 13, 2764. 10.3390/nu13082764 34444924 PMC8399049

[nbu12571-bib-0031] OECD/European Union (2018) Health at a Glance: Europe 2018. Available at: https://www.oecd‐ilibrary.org/docserver/health_glance_eur‐2018‐en.pdf?expires=1656523703&id=id&accname=guest&checksum=B38439C5167F262937B7392C39206095. [Accessed 17 March 2022].

[nbu12571-bib-0032] Olofsson, J.K. , Ekström, L.M. & Nordin, S. (2021) Olfaction and aging: A review of the current state of research and future directions. I‐Perception, 12(3), 20416695211020331. 10.1177/20416695211020331 34249327 PMC8239976

[nbu12571-bib-0033] Pivi, G.A.K. , V da Silva, R. , Juliano, Y. , Novo, N.F. , Okamoto, I.H. , Brant, C.Q. et al. (2011) A prospective study of nutrition education and oral nutritional supplementation in patients with Alzheimer's disease. Nutrition Journal, 10(1), 1–6. 10.1186/1475-2891-10-98 21943331 PMC3189102

[nbu12571-bib-0034] Poey, J. L. , Burr, J. A. & Roberts, J. S. (2017) Social connectedness, perceived isolation, and dementia: Does the social environment moderate the relationship between genetic risk and cognitive well‐being? Gerontologist, 57(6), 1031–1040. 10.1093/geront/gnw154 28329797

[nbu12571-bib-0035] Prince, M. , Guerchet, M. , Albanese, E. & Prina, M. (2014) Nutrition and dementia: A review of available research. London, UK: Alzheimer's Disease International. http://www.alz.co.uk/nutrition‐report [Accessed 25 January 2022].

[nbu12571-bib-0036] Prinelli, F. , Adorni, F. , Leite, M. , Pettenati, C. , Russo, A. , Di Santo, S. et al. (2018) Different exposures to risk factors do not explain the inverse relationship of occurrence between cancer and neurodegenerative diseases: an Italian nested case‐control study. Alzheimer Disease and Associated Disorders, 32(1), 76–82. 28796009 10.1097/WAD.0000000000000204

[nbu12571-bib-0037] Prinelli, F. , Fratiglioni, L. , Musicco, M. , Johansson, I. , Adorni, F. , Shakersain, B. et al. (2019) The impact of nutrient‐based dietary patterns on cognitive decline in older adults. Clinical Nutrition, 38(6), 2813–2820. 10.1016/j.clnu.2018.12.012 30591381

[nbu12571-bib-0038] Prinelli, F. , Jesuththasan, N. , Severgnini, M. , Musicco, M. , Adorni, F. , Leite, M. et al. (2020) Exploring the relationship between nutrition, gUT microbiota, and BRain AgINg in community‐dwelling seniors: The Italian NutBrain population‐based cohort study protocol. BMC Geriatrics, 20(1), 1–11. 10.1186/s12877-020-01652-2 PMC7376643 32703186

[nbu12571-bib-0039] Richard, C. , Couture, P. , Desroches, S. & Lamarche, B. (2013) Effect of the mediterranean diet with and without weight loss on markers of inflammation in men with metabolic syndrome. Obesity, 21(1), 51–57. 10.1038/oby.2012.148 23505168

[nbu12571-bib-0040] Ronveaux, C.C. , Tomé, D. & Raybould, H.E. (2015) Glucagon‐like peptide 1 interacts with ghrelin and leptin to regulate glucose metabolism and food intake through vagal afferent neuron signaling. Journal of Nutrition, 145(4), 672–680. 10.3945/jn.114.206029 25833771 PMC4381768

[nbu12571-bib-0041] Salva, A. , Andrieu, S. , Fernandez, E. , Schiffrin, E.J. , Moulin, J. , Decarli, B. et al. (2011) Health and nutrition promotion program for patients with dementia (NutriAlz): Cluster randomized trial. Journal of Nutrition, Health and Aging, 15(10), 822–830. 10.1007/s12603-011-0363-3 22159768

[nbu12571-bib-0042] Sanders, C.L. , Wengreen, H. , Schwartz, S. , Behrens, S.J. , Corcoran, C. , Lyketsos, C.G. et al. (2018) Nutritional status is associated with severe dementia and mortality: The cache county dementia progression study. Alzheimer Disease & Associated Disorders, 32(4), 298–304. 30188355 10.1097/WAD.0000000000000274 PMC6345543

[nbu12571-bib-0043] Scarmeas, N. , Stern, Y. , Tang, M.X. , Mayeux, R. & Luchsinger, J.A. (2006) Mediterranean diet and risk for Alzheimer's disease. Annals of Neurology, 59(6), 912–921. 10.1002/ana.20854 16622828 PMC3024594

[nbu12571-bib-0044] Sergi, G. , De Rui, M. , Coin, A. , Inelmen, E.M. & Manzato, E. (2013) Weight loss and Alzheimer's disease: Temporal and aetiologic connections. Proceedings of the Nutrition Society, 72(1), 160–165. 10.1017/S0029665112002753 23110988

[nbu12571-bib-0045] Soto, M.E. , Secher, M. , Gillette‐Guyonnet, S. , van Kan, G.A. , Andrieu, S. , Nourhasemi, F. et al. (2012) Weight loss and rapid cognitive decline in community‐dwelling patients with Alzheimer's disease. Journal of Alzheimer's Disease, 28(3), 647–654. 10.3233/JAD-2011-110713 22045479

[nbu12571-bib-0046] Spaccavento, S. , Del Prete, M. , Craca, A. & Fiore, P. (2009) Influence of nutritional status on cognitive, functional and neuropsychiatric deficits in Alzheimer's disease. Archives of Gerontology and Geriatrics, 48(3), 356–360. 10.1016/j.archger.2008.03.002 18448178

[nbu12571-bib-0047] Tosti, V. , Bertozzi, B. & Fontana, L. (2018) Health benefits of the Mediterranean diet: metabolic and molecular mechanisms. The Journals of Gerontology: Series A, 73(3), 318–326. 10.1093/gerona/glx227 PMC7190876 29244059

[nbu12571-bib-0048] Trommelen, J. , Betz, M.W. & van Loon, L.J.C. (2019) The muscle protein synthetic response to meal ingestion following resistance‐type exercise. Sports Medicine, 49(2), 185–197. 10.1007/s40279-019-01053-5 30659499

[nbu12571-bib-0049] Vanhanen, M. , Kivipelto, M. , Koivisto, K. , Kuusisto, J. , Mykkänen, L. , Helkala, E.L. et al. (2001) APOE‐ε4 is associated with weight loss in women with AD: A population‐based study. Neurology, 56(5), 655–659. 10.1212/WNL.56.5.655 11245719

[nbu12571-bib-0050] Vicente de Sousa, O. , Soares Guerra, R. , Sousa, A.S. , Pais Henriques, B. , Pereira Monteiro, A. & Amaral, T.F. (2017) Impact of nutritional supplementation and a psychomotor program on patients with Alzheimer's disease. American Journal of Alzheimer's Disease and Other Dementias, 32(6), 329–341. 10.1177/1533317517705221 PMC10852851 28446028

[nbu12571-bib-0051] Volkert, D. , Chourdakis, M. , Faxen‐Irving, G. , Frühwald, T. , Landi, F. , Suominen, M.H. et al. (2015) ESPEN guidelines on nutrition in dementia. Clinical Nutrition, 34(6), 1052–1073. 10.1016/j.clnu.2015.09.004 26522922

[nbu12571-bib-0052] World Health Organization (2019) WHO Clinical Consortium on Healthy Ageing 2019, World Health Organisation: Clinical consortium on healthy ageing. Available at: https://www.who.int/publications/i/item/9789240009752#:~:text=. The 2019 annual meeting of the World Health, everyone can live a long and healthy life. [Accessed 25 January 2022].

[nbu12571-bib-0053] World Health Organization (WHO) . (2017) ‘Global action plan on the public health response to dementia 2017–2025’. Geneva: World Health Organization, p. 27. Available at: http://www.who.int/mental_health/neurology/dementia/action_plan_2017_2025/en/. [Accessed 25 January 2022].

[nbu12571-bib-0054] Wouters‐Wesseling, W. , Wagenaar, L.W. , Rozendaal, M. , Deijen, J.B. , deGroot, L.C. & Bindels, J.G. (2005) The effect of supplementation with an enriched drink on indices of immune function in frail elderly. Journal of Nutrition, Health and Aging, 9(4), 281–286. 15980931

